# A new method for testing non-porous surfaces for their antimicrobial efficacy using an aerosol-generating spray chamber

**DOI:** 10.3389/fmicb.2024.1508596

**Published:** 2025-01-07

**Authors:** Sabine Poelzl, Daniela Dreisiebner, Eva Zarschenas, Rozita Nokhbehzaeim, Clemens Kittinger

**Affiliations:** Diagnostic and Research Institute for Hygiene, Microbiology and Environmental Medicine, Medical University of Graz, Graz, Austria

**Keywords:** antimicrobial non-porous surface, ISO 22196:2011, ISO 7581:2023, aerosol, spraying chamber, CAMAG

## Abstract

The application of antimicrobial surfaces requires proof of their effectivity by *in vitro* methods in laboratories. One of the most common test methods is ISO 22196:2011, which represents a simple and inexpensive protocol by applying the bacterial suspension with known volume and concentration covered under a polyethylene film on the surfaces. The incubation is then conducted under defined humidity conditions for 24 h. Another approach for testing non-porous surfaces is the newly published ISO 7581:2023. With this protocol, a “dry test” is achieved by spreading and drying 1 μL of a bacterial suspension on the surfaces. A comprehensive evaluation of both standard protocols was conducted. This showed that they have some limitations and often do not include realistic test conditions that refer to the final product. Accordingly, the objective of this study was to develop a novel testing procedure that uses the spraying of a suspension inside of a chamber to generate aerosols with a precisely defined bacterial or yeast load. The samples to be analyzed are covered with small droplets that dry up within a few minutes and thus enable very reproducible contamination of the surfaces. The test series was carried out with low-alloyed carbon steel and glass without antimicrobial substances against two different *Escherichia coli* and *Staphylococcus epidermidis* strains and one *Candida albicans* strain to evaluate the new method. The results provided reproducible and reliable results in the setup carried out. This test method represents a valuable alternative for the assessment of non-porous surfaces in a manner that more closely reflects real-world conditions (e.g., simulation of aerosol formation by sneezing).

## 1 Introduction

The spread of pathogens via contaminated surfaces is a major challenge for hygiene management. One promising approach to minimize the propagation of these organisms is the use of antimicrobial coatings (AMCs) or antimicrobial materials (AMMs) in general. Many different materials have already been investigated for their antimicrobial efficacy. The materials can be divided into three groups depending on the mechanisms of action: active substance-release AMMs, potentiated surface-based AMMs (including biocides, metals, peptides, or amines on their surfaces), and non-adhesive AMMs (Campoccia et al., 2013; Sjollema et al., 2018; Cunliffe et al., 2021). The metal most commonly used as AMC is copper, which can kill at least 7 to 8 logs of microorganisms per hour (Grass et al., 2011). Furthermore, zinc and combinations of different metals can also be part of AMCs (Garza-Cervantes et al., 2017; Poelzl et al., 2024). In addition to metals, chemicals, plant extracts, enzymes, and bacteriocins are also used as antimicrobial substances (Fadiji et al., 2023). A summary of antimicrobial substances and their effects was recently published by Kışla et al. (2023).

Before these AMMs or AMCs can be sold on the market and used as antimicrobial surfaces, they need to be extensively tested by *in vitro* methods in laboratories to prove their effectiveness. One of these test methods is ISO 22196:2011 (International Organization for Standardization, 2011), which represents a simple and inexpensive protocol by applying the bacterial suspension with known volume and concentration covered under a polyethylene film on the test surfaces. The incubation is then conducted under defined humidity conditions for 24 h. Even though the protocol seems reliable and easy to follow, there are some consistently discussed critical points of ISO 22196:2011. The most important points are the incubation condition (duration, temperature, and relative humidity) as well as the unrealistic type of testing, as the test bacteria are in contact with an active ingredient over a liquid phase (Wiegand et al., 2018; Cunliffe et al., 2021). A new test method has recently been published to improve the current protocol to make the test more applicable in practice. ISO 7581:2023 (International Organization for Standardization, 2023) offers a “dry test” by spreading and drying 1 μL of a bacterial suspension on the surfaces. A detailed comparison of the two ISO protocols has already been undertaken in a previous study (Maitz et al., 2024). However, both protocols have some shortcomings. Briefly summarized, the application of the bacterial suspension under a polyethylene film (ISO 22196:2011) does not reflect real-life conditions, while pipetting and distributing 1 μL (ISO 7581:2023) is difficult and leads to inaccuracies (e.g., different drying times on each testing surface). The incubation time of 24 h stated by ISO 22196:2011 does often not allow an interpretation as false-positive results for the reference material can be generated (Gram-negative bacteria usually die off, after 24 h). The different *E. coli* strains used in the two standard protocols should actually show the same sensitivity, but even the small difference in the bacterial strains leads to different results in antimicrobial efficacy. Neither of these two test protocols is robust and application-oriented, and both need an adjustment in the application of the bacterial suspension and more customization options when it comes to incubation conditions and test organisms. In this study, a new testing procedure was used that involves spraying bacterial/yeast suspensions inside a chamber to produce aerosols to simulate more realistic test conditions (e.g., simulation of aerosol formation by sneezing or coughing). The tested samples are thus covered with small droplets that dry up within a few minutes and allow a timely continuation of the experiments (without a massive loss of viability of the bacterial strains due to drying effects). Furthermore, this study again demonstrates the importance of selecting the correct microbes and conditions for the chosen test method to reflect the conditions under which the surface is to be used. The aim of this study was to evaluate the spraying process to obtain knowledge on the practicability and reproducibility of the method.

## 2 Materials and methods

The workflow was established by adapting ISO 22196:2011 measurement of antibacterial activity on plastics and other non-porous surfaces (International Organization for Standardization, 2011) and ISO 7581:2023 evaluation of the bactericidal activity of a non-porous antimicrobial surface used in a dry environment (International Organization for Standardization, 2023) and adding the spray chamber CAMAG Derivatizer Base Unit as a device to apply the bacterial/yeast suspension on the samples.

### 2.1 Samples

Two non-porous samples were analyzed in this study: low-alloyed carbon steel (voestalpine Stahl GmbH, Linz, Austria) and glass (slide 50 × 50 × 1.55 mm, Cloeren Technology GmbH, Wegberg, Germany), both without an antimicrobial agent. All samples had the same size of 50 × 50 mm. In the microbiological laboratory, the samples were stored at room temperature. Prior to testing, the samples were disinfected with 70% ethanol (Merck KGaA, Darmstadt, Germany).

### 2.2 Strains and preparation of the bacterial/yeast suspensions

Five different strains were used for the testing procedure: *Escherichia coli* (*E. coli*) DSM 1576 (specified by ISO 22196:2011), *E. coli* DSM 682 (specified by ISO 7581:2023), *Staphylococcus epidermidis* (*S. epidermidis*) DSM 1798, *S. epidermidis* DSM 3269, and *Candida albicans* (*C. albicans*) DSM 1386 (Leibniz Institute DSMZ—German Collection of Microorganisms and Cell Cultures GmbH, Braunschweig-Sued, Germany). The *E. coli* strains were selected according to the ISO specifications, while *C. albicans* was used according to “Deutsche Gesellschaft für Hygiene und Mikrobiologie e.V.” (Deutsche Gesellschaft Für Hygiene und Mikrobiologie, 2022). For the selection of Gram-positive bacteria, testing against the usually specified *Staphylococcus aureus* strains has so far been avoided for safety reasons, and “more harmless” representatives with less pathogenicity factors of the same genus have been chosen. The strains were cultivated overnight (16–20 h) on Columbia Blood Agar plates (Becton Dickinson GmbH, Heidelberg, Germany) at 36°C ± 2°C or 30°C ± 2°C (only *C. albicans*). The cell material was inoculated in 0.2% v/v Tryptone Soy Broth (TSB, Oxoid Limited, Hampshire, United Kingdom) diluted in distilled water. A VITEK^®^ DensiCHEK device (bioMerièux Austria GmbH, Vienna, Austria) was used to obtain a bacterial/yeast solution with 10^8^ colony-forming units (CFUs)/mL. To obtain an initial concentration of 2.5 × 10^5^–10 × 10^5^ CFU/mL according to the two ISO protocols, the bacterial/yeast suspension was diluted accordingly in 0.2% v/v TSB. To determine the concentration of the test suspension, the adjusted bacterial/yeast suspensions were serially diluted with 1X phosphate-buffered saline (PBS, Carl Roth GmbH + Co. KG, Karlsruhe, Germany). After incubation of 500 μL of those solutions for 24 h at 36°C ± 2°C or for 48 h at 30°C ± 2°C (only *C. albicans*) on tryptic soy agar (TSA, VWR International GmbH, Darmstadt, Germany) plates, plates from one dilution containing 30–300 CFU were selected and counted. The weighted mean bacterial/yeast concentration was calculated in CFU with the following formula:


X=C∗D

where *X* is the initial suspension concentration (applied load) in CFU, *C* is the average plate count for the duplicate plates, and *D* is the dilution factor for the plates counted.

### 2.3 Testing procedure

**Figure 1:**
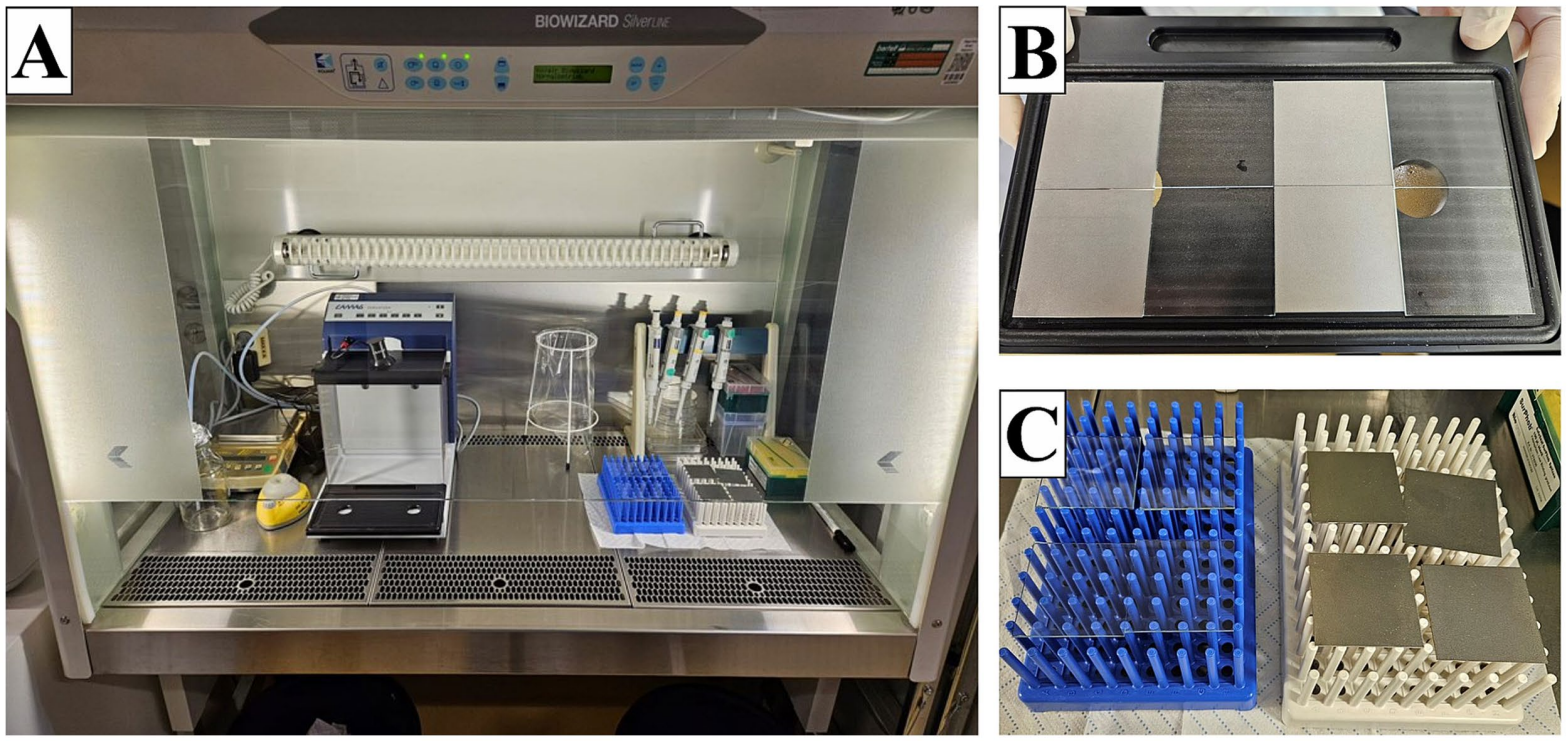
Setup for the spraying process under the laminar flow. **(A)** Entire experimental setup. **(B)** Eight samples after the spraying process. **(C)** Drying of the samples on plastic racks.

The entire process of spraying and drying the samples was carried out under a laminar flow (Kojair Tech Oy, Mänttä-Vilppula, Finland) (Figure 1A). Each specimen was placed on the plate of the spray chamber CAMAG Derivatizer Base Unit (CAMAG^®^ Derivatizer, Muttenz, Switzerland) with the test surface facing upward. Then, the plate carrying a total of eight samples was inserted into the device, and the hood was closed. Next, 500 μL of the bacterial/yeast suspension with an expected concentration of 2.5 × 10^5^–10 × 10^5^ CFU/mL was pipetted into the red or yellow nozzle (used for high viscosity of the spray reagent), and the whole volume was sprayed in the process. The spraying time was set on level 4 (middle spraying speed). After filling the bacterial/yeast suspension in the nozzle, the spraying process was started immediately to avoid unwanted dripping. With this setting, the spraying process took approximately 45 s, followed by a pause of 2 min during which the aerosol should settle. The pumping process of the device then took another 1 min. The hood was again lifted, and the plate with the samples was removed (Figure 1B). The samples were transferred to a sterilized plastic rack for drying (Figure 1C). The drying time was a maximum of 6 min 30 s. This results in a total incubation of the MOs on the samples of approximately 10 min before the samples are further processed, assuming that the spraying time was maintained (the spraying time can be longer with older nozzles). After drying, the samples for 0 h were transferred into a plastic container with 10 mL of a neutralizer called soybean casein digest broth with lecithin and polyoxyethylene sorbitan monooleate (SCDLP broth) containing TSB (Oxoid Limited, Hampshire, United Kingdom), lecithin (Carl Roth GmbH + Co. KG, Karlsruhe, Germany), and Tween^®^ 80 (Amresco Inc., Solon, Ohio, United States) as well as 12 g–14 g glass beads (2.85–3.3 mm, Carl Roth GmbH + Co. KG, Karlsruhe, Germany) with the test surface facing downward to rescue surviving bacteria. The other samples for further incubation (1 h) were each transferred into a sterile Petri dish (90 mm × 16.2 mm, Fisher Scientific, Schwerte, Germany) with the test surface facing upward. The incubation was performed at 20°C ± 1°C with relative humidity (RH) between 30 and 65% for 1 h as recommended by ISO 7581:2023. After 1 h, these samples were also transferred into the plastic container with neutralizer and glass beads. The samples were shaken in the recovery liquid for 3 min at 200 rpm on a Battery Shaker KM 2 Akku (Edmund Bühler GmbH, Bodelshausen, Germany). The dilution series were performed in 1X PBS. Finally, 500 μL of appropriate dilutions were plated on TSA plates in duplicates by the spread plate technique. All plates with 30–300 CFU after incubation for 24 h or 48 h at 36°C ± 2°C or at 30°C ± 2°C were counted.

The applied load is defined as the actual number of bacterial or yeast cells sprayed onto the specimens in the experiment. Time point 0 h is defined as the test point where the bacterial/yeast suspension is completely dry on the surface and harvested immediately to ensure the initial concentration of each sample and validate loss due to manipulation. For each incubation time, duplicates (*n* = 2) of each sample type were used in three independent runs (*n* = 6) to calculate the mean and standard deviation for antibacterial activity. When no colonies were countable on the plates, the limit of detection was set at 10 CFU since 10 mL of neutralization medium was used.

For each test sample, the recovered number of viable bacteria in CFU was calculated using the following formula:


N=C∗V∗D

where *N* is the number of viable bacteria recovered per test specimen (CFU), *C* is the average plate count for the duplicate plates, *V* is the volume, in ml, of SCDLP added to the specimen (10 mL), and *D* is the dilution factor for the plates counted.

The calculation of the reduction was conducted with the following formula:


$$R=\frac{\left(U_{0}-U_{t}\right)}{U_{0}}\;\mathrm{or}\;R=\frac{U_{0}}{U_{t\text{.}}}$$

where *R* is the antibacterial activity (%) or (log_10_), *U*_0_ is the average of the common logarithm of the number of viable bacteria recovered from the test specimens immediately after inoculation (0 h), and *U*_t_ is the average of the common logarithm of the number of viable bacteria recovered from the test specimens after 1 h.

The verification of the methodology was calculated through 0-h replicates (*n* = 6) with the following formula:


$$\frac{\left(L_{\text{max}}-L_{\text{min}}\right)}{L_{\mathrm{mean}}}\leq 0.2$$

where *L*_max_ is 10 logarithm of the maximum number of viable bacteria found on a specimen, *L*_min_ is 10 logarithm of the minimum number of viable bacteria found on a specimen, and *L*_mean_ is 10 logarithm of the mean number of viable bacteria found on the specimens. A value ≤0.2 indicated a valid test result.

To have another verification of this protocol, the loss of bacteria during the spraying process was calculated. Therefore, for every independent run, the loss of applied load was calculated as follows:


$$\frac{\left(\frac{X}{2}\right)}{S_{0}}\leq 0.5\;\log_{10}$$

where *X* is the initial suspension concentration (applied load) in CFU (divided by 2, since only half of the samples (=0 h) of one run were included) and *S*_0_ is the sum of the common logarithm of the number of viable bacteria recovered from the test specimens immediately after inoculation (0 h)

The average of the three independent runs was calculated, and a value ≤0.5 log_10_ showed a low loss of the test organism and therefore a good initial concentration or an appropriate strain for the experiment. In addition, the incubation time and the sprayed volume can also be monitored with this value. If the spraying process takes too long and/or the entire volume cannot be spayed, which is often caused by an outdated nozzle, the difference between the applied load and the results of 0 h can also increase.

### 2.4 Data analysis

The results were expressed as described with corresponding mean ± standard deviation (SD). Depictions were generated using CorelDRAW 2019 (Corel Corporation, Ottawa, Canada) and GraphPad Prism Version 10 (GraphPad Software, Boston, MA, United States).

## 3 Results

The low-alloyed carbon steel and glass samples were tested against five different strains to obtain and evaluate the new testing strategy with the nebulizer of CAMAG^®^ Derivatizer.

**Figure 2:**
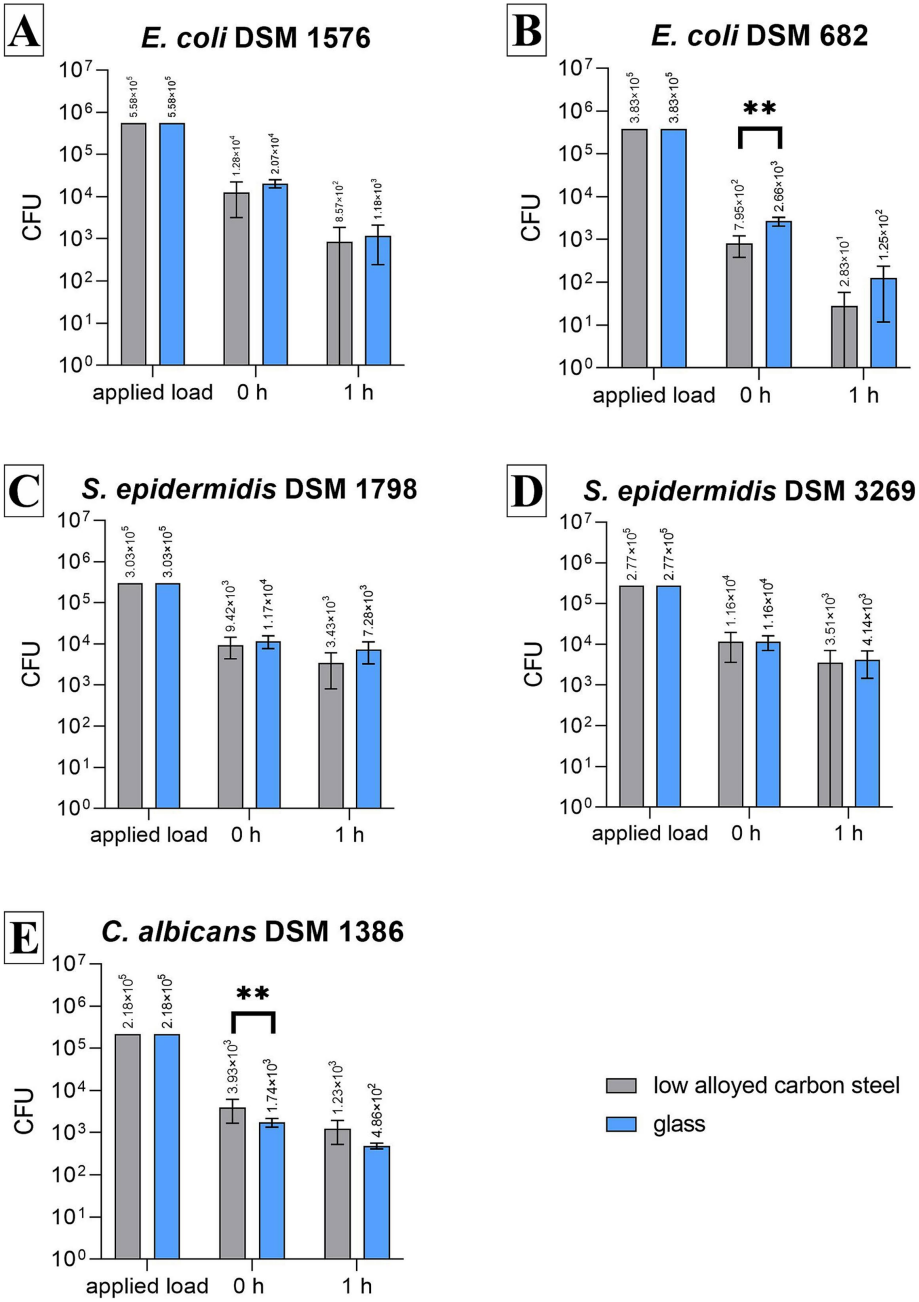
Results of all tested strains using the testing procedure in a CAMAG Derivatizer Base Unit. 500 μL of bacterial/yeast suspensions with 2.5 × 10^5^–10 × 10^5^ CFU/mL of *E. coli* DSM 1576 **(A)**, *E. coli* DSM 682 **(B)**, *S. epidermidis* DSM 1798 **(C)**, *S. epidermidis* DSM 3269 **(D)**, or *C. albicans* DSM 1386 **(E)** were sprayed over eight samples (four low-alloyed carbon steel samples and four glass samples), respectively. The temporal incubation (1 h) was performed at 20°C ± 1°C and RH of 30%–65%. Furthermore, 0 h shows recovery immediately after the suspension has dried on the surface. After the time points, the bacteria were harvested and checked for survival. The error bars indicate the standard errors of the respective means, which were composed of duplicates in three independent runs (*n* = 6). The limit of detection was set at 10 CFU. Statistically significant differences between low-alloyed carbon steel and glass within the same incubation time are marked (mean with 95% CI; Mann–Whitney *U*-test; *p*-value: <0.05; ^**^indicates statistical significance with a *p*-value below 0.0021).

Both strains of *S. epidermidis* remained reasonably stable and detectable on low-alloyed carbon steel and glass after 1 h compared to 0 h (Figures 2C,D) as the reduction for Gram-positive bacteria was below 1 log_10_. Compared to the 0-h results, a 37.75% to approximately 70% reduction was calculated (Table 1). These results were expected as no antimicrobial substances were used in this study. In contrast, both *E. coli* strains showed a reduction after 1 h. Especially, *E. coli* DSM 682 decreased rapidly with 2.5 log_10_ (low-alloyed carbon steel) and 2.1 log_10_ (glass) within the drying time (0 h). A further decrease of 1.4 log_10_ (low-alloyed carbon steel) and 1.2 log_10_ (glass) was detectable between 0 h and 1 h (Figure 2B and Table 1). The decrease for the other *E. coli* strain (DSM 1576) showed similar results between 0 h and 1 h (reduction of approximately 1.1 log_10_, Table 1). However, *E. coli* DSM 1576 remained more stable on the surfaces after drying time (0 h) (Figure 2A). It can be concluded that *E. coli* DSM 682 does not survive as well as *E. coli* DSM 1576 in dry conditions. The yeast *C. albicans* DSM 1386 showed similar 0 h results as *E. coli* DSM 682. A loss of 1.11 log_10_ compared to the applied load was calculated for the yeast, while *E. coli* DSM 682 had a loss of 1.28 log_10_. The other microorganisms were all beneath the set threshold of 0.5 log_10_ (Table 1). Even though the drying process in the beginning affected *C. albicans* DSM 1386, the yeast remained quite stable for the incubation period of 1 h. Only a negligible reduction of <1 log_10_ was detectable between 0 h and 1 h on low-alloyed carbon steel and glass surfaces (Figure 2E and Table 1).

**Table 1:** Reduction of the tested strains after 1 h of incubation on the tested specimens, test validity, and loss of applied load during the spraying process.

	Low-alloyed carbon steel			Glass			
	%	log_10_	Test validity	%	log_10_	Test validity	Loss of applied load
*E. coli* DSM 1576	93.27	1.15	0.10	94.32	1.18	0.06	0.44 log_10_
*E. coli* DSM 682	97.27	1.37	0.18	95.42	1.22	0.08	1.28 log_10_
*S. epidermidis* DSM 1798	63.58	0.28	0.16	37.75	0.16	0.09	0.39 log_10_
*S. epidermidis* DSM 3269	69.78	0.33	0.23	64.40	0.28	0.11	0.41 log_10_
*C. albicans* DSM 1386	68.67	0.32	0.15	72.12	0.36	0.07	1.11 log_10_

The decrease after 1 h was calculated in percentage or log_10_ by the results of 0 h on each sample of low-alloyed carbon steel or glass. For the results of the test validity, all values (*n* = 6) of the three independent runs were considered. High variances were present between individual runs, which is why the test validity values are also high. However, individual runs had all values ≤0.2. The loss of bacteria through the spraying process in the chamber was calculated for each independent run, and the average value is shown.

The difference between the microorganisms can be seen when all results are compared with one type of surface. At all time points tested, the least number of recovered cells was either countable for *E. coli* DSM 682 or *C. albicans* DSM 1386. The other three bacteria strains achieved approximately the same results. The efficacy between the test surfaces of low-alloyed carbon steel and glass was approximately equal. This was expected since no antimicrobial agents were added to these surfaces and no antimicrobial efficacy should be detected. Significant differences (*p*-value <0.05) were only calculated for *E. coli* DSM 682 and *C. albicans* at 0 h.

## 4 Discussion

ISO 22196:2011 has often been discussed in the literature, and its approach to test antimicrobial non-porous surfaces has been challenged (Wiegand et al., 2018; Cunliffe et al., 2021; Bäumler et al., 2022; Bento de Carvalho et al., 2024; Maitz et al., 2024). This international standard does not allow any modifications to the incubation conditions (duration, temperature, or humidity). In addition, there are no alterations to the application of the bacterial suspension. It is generally unfeasible to conduct realistic tests on the utilization of the end product in accordance with these rigorous specifications. Therefore, some researchers already focused on optimizing the testing protocol, for instance, using the large-droplet inoculation (LDI) method (Caschera and Lukasz, 2016; Caschera et al., 2021; Kaur et al., 2023) or a touch transfer assay (Knobloch et al., 2017). Moreover, another focus was placed on spraying techniques to mimic aerosol formation, e.g., when sneezing or coughing. An example of a study with aerosol formation was developed by Ojeil et al. (2013). The researchers initially assessed the environmental conditions (RH, temperature, and soiling) in a hospital and then used these parameters to test copper alloys against *Staphylococcus aureus* with a nebulizer, which was connected to a cascade impactor. Building on this study, McDonald et al. (2020) also used a nebulizing test arrangement to achieve a realistic deposition of the bacterial inoculum to the test surface. Since the application of the bacterial suspension by a nebulizing process seems to be the most realistic method for us, a setup using a CAMAG Derivatizer Base Unit was tested. This device is normally used for chromatography to spray different chemical reagents onto TLC/HPTLC plates. In a few studies, however, bacterial strains have also been successfully used in this spraying chamber, for instance, to improve the evaluation of SOS-UMU-C assays (Mehl and Morlock, 2023; Windisch et al., 2024). Preliminary tests showed that a volume of 500 μL is sufficient to moist the eight samples homogenously but not too much so that the liquid can still dry quickly and thus provide a good initial basis for testing antimicrobial surfaces. It was also found that a concentration of 10^5^ CFU/mL as specified in ISO 22196:2011 (2.5 × 10^5^–10 × 10^5^ CFU/mL) can be used. Moreover, the applied concentration is also comparable with the new testing method of ISO 7581:2023. The main difference, however, is that the 10^5^ CFU/mL are applied/sprayed simultaneously on eight testing surfaces and not one. This is also the reason for the significant difference between the applied load and 0 h, given that the quantity of sprayed bacteria/yeast cells is distributed across eight samples. Testing this setup repeatedly, a difference of less than 0.5 log_10_ between the applied load and the sum of 0 h results for each spraying cycle was calculated. The only exception with 2.82 × 10^1^ CFU was *E. coli* DSM 682 and *C. albicans* with 1.10 × 10^1^ CFU. Both strains showed also a significant difference at 0 h between the two surfaces tested. It can be concluded that both strains are suitable for this test method, although with reservations, given that the bacterial/yeast concentration decreases during the drying process even in the absence of a biocide. The decrease in concentration was therefore due to the drying process on the surfaces which mainly affects Gram-negative (as a matter of their cell wall structure) strains. The loss of *C. albicans* is due to the reduced permeability of the nozzle for the larger *Candida* cells. In addition, all spraying processes have to deal with condensation on the chamber walls. To speed up the drying process or to achieve a uniform drying duration, it is necessary to remove the samples from the chamber after the spraying is completed and place them on a rack where the airflow can reach them easily from all sides. Otherwise, the drying will take too long or will be inconsistent (some samples might be dry, while others will be still moist). As summarized by Bäumler et al. (2022), the drying depends on the respective surface material and can take seconds but also up to minutes. Therefore, optimization is necessary to minimize the differences. Furthermore, the incubation was conducted at 30 to 65% and 20°C (based on the specification of ISO 7581:2023) because an intermediate RH and room temperature is closer to realistic environmental conditions for most applications than 37°C and >90% RH as specified by ISO 22196:2011. The test procedure that has been established produces results that are reproducible, which can be achieved quickly and straightforwardly.

As mentioned above, *E. coli* DSM 682 showed major differences to the other strains of *E. coli*, although according to ISO 22196:2011 and ISO 7581:2023, these strains should be used. These results are also consistent with the comparison of these two standardized protocols, where *E. coli* DSM 682 could also not survive long on the tested surfaces (Maitz et al., 2024). Both show the same multiple antibiotic resistances against antibiotics that have no effect against Gram-negative bacteria anyway (e.g., vancomycin, lincomycin, or oxacillin). *E. coli* DSM 1576 was isolated from feces back in 1979, while *E. coli* DSM 682 was collected from an unknown source before 1965 (Reimer et al., 2022). Why both ISOs did not select identical strains for testing remains questionable to us. This underlines again the requirement for an appropriate selection of test bacteria, conditions, and application techniques as well as reference material (Kaur et al., 2023), to achieve robust and reliable data that are comparable within the experiments.

Due to safety concerns, microorganisms with a high pathogenicity factor were excluded from the initial evaluation of the new testing method. All microorganisms tested are classified as BSL-1 organisms by the ATCC. In addition, *S. epidermidis* strains were chosen instead of *Staphylococcus aureus* which is required by the ISO protocols because *S. epidermidis* does not cause pneumonia. Moreover, the first step in this type of test procedure was also to ensure the biosafety of such a chamber, which has been proven so far. As the manufacturer does not guarantee the complete tightness of the aerosol chamber, we used sedimentation plates that showed 99% no bacterial growth, and we utilized laminar flow as an additional security measure. As this study was only intended to test the novel test system in the aerosol chamber, further investigations can be conducted with additional strains in due course.

To conclude, the new testing procedure with the nebulizer chamber from CAMAG^®^ Derivatizer generated reproducible and reliable results in the setup carried out, with simple and quick application at the same time. It is therefore recommended that this testing procedure be used as an effective alternative for the assessment of non-porous surfaces under conditions that more accurately reflect real-world scenarios.

## Data Availability

The original contributions presented in the study are included in the article/supplementary material, further inquiries can be directed to the corresponding author.
